# Do different surgical techniques in tibia pilon fractures change the results of the midterm?

**DOI:** 10.3906/sag-2006-212

**Published:** 2020-10-22

**Authors:** Vedat BİÇİCİ, İzzet BİNGÖL

**Affiliations:** 1 Department of Orthopaedics and Traumatology, Ankara City Hospital, Ankara Turkey; 2 Department of Orthopaedics and Traumatology, 29 Mayıs Goverment Hospital, Ankara Turkey

**Keywords:** Pilon fracture, distal tibia, external fixation, Ilizarov external fixator, AOFAS

## Abstract

**Background/aim:**

Pilon fracture is difficult to treat fractures due to many complications that can develop after surgery. To achieve the best results, different surgical approaches are used.In our study, we aimed to compare the functional results and complication rates of our treatments in patients treated with 3 different surgical tecniques.

**Materials and methods:**

89 pilon fractures of 87 patients treated for pilon fracture were evaluated. Patients were examined in 3 different groups (one step, two step surgery and Ilizarov). Functional results, postoperative complications and ankle AOFAS scores were evaluated.

**Results:**

The mean AOFAS score of the all patients was 77.67. There was no significant difference between 3 surgical techniques (P = 0,880). While skin complication was not seen in patients who underwent double-stage surgery and Ilizarov (0%); It was seen in 6 (15.7%) patients who underwent single-stage surgery. Treatment results were found to be better in type 1 and type 2 fractures, while in type 3 fractures (P = 0.004).

**Conclusion:**

Despite the different surgical approaches and implants applied, no difference was found between the midterm ankle functional results of the patients. Two-stage surgery and Ilizarov is a safe and effective treatment approach to reduce morbidity and early complications in pilon fractures.

## 1. Introduction

Tibia pilon fractures; fractures of the tibia involving the distal metafizer region and the joint surface [1]. Its frequency is gradually increasing due to prolonged life span, traffic accidents, widespread sports activities, falling from height. Pilon fractures, which are more common especially in the fourth decade and in men, constitute 1% of all lower limb fractures and 7%–10% of all tibial fractures [2]. Due to the low incidence of pilon fractures, the fragmentation of the metafizer region and the joint due to high energy mechanisms, chondral injuries, deep injuries and compartment syndrome, it is one of the fractures that forced orthopedists most late and gained late experience [3].

The ideal treatment method for pilon fractures is still controversial. The main purposes in treatment are protecting soft tissues, ensure proper alignment, restore joint surface, reduce complications such as wound healing and infection [4]. For this reason, open reduction and internal fixation (ORIF) are most frequently used in treatment. However, due to the widespread tissue edema especially in the early phase of the fracture, extensive soft tissue dissection postoperative wound healing problems increase the frequency of infection [5]. In addition, disruption of the distal tibia due to fracture and surgery can lead to broken union problems [6]. For these reasons, different treatment methods such as open reduction internal fixation, external fixation in the acute phase and subsequent application of ORIF, Ilizarov are applied, in order to reduce complications in treatment and to better manage fracture healing. However, different results are reported depending on these treatments [7–8]. Due to all these difficulties and differences in treatment approaches, in this retrospective study, we aimed to evaluate the midterm functional and clinical results of our patients who underwent different surgical methods due to pilon fracture in our clinic and to compare the treatment approaches among themselves.

## 2. Materials and methods

### 2.1. Study design and participants

After obtaining the necessary ethics committees and permits, the files, radiology and outpatient records of the patients who applied to our clinic were evaluated retrospectively. 89 pilon fractures of 87 patients who were applied and operated due to pilon fracture were evaluated. The exclusion criterion was determined as open fractures other than the Gustilo–Anderson type 1 open fracture, pediatric fractures under the age of 18, stress fractures, pathological fractures, segmental fractures, patients who had undergone ankle surgery before, did not follow regularly and did not want to participate in the study. In order to better understand fracture morphology and pathology, all patients underwent 2-way ankle radiographs and ankle computed tomography (CT). All patients were classified according to the Rüedi/Allgöwer [9] classification, and patients with open fractures were classified according to the Gustilo–Anderson [10] classification. Postoperative radiological imaging, early complications such as superficial or deep infection and late complications such as union delay, nonunion, impaired failure, late infection, osteomyelitis were evaluated in our patients. Functional ankle results of the patients were evaluated with American Ankle Scoring (AOFAS) [11]. According to AOFAS score, 0–69 points are poor, 70–79 points are mild, 80–89 points are good, 90–100 points are excellent.

## 2.2. Surgical technique

The patients who applied to our hospital with pilon fracture were closed reduction and splint in the emergency room and the limb was stabilized. After debridement and abundant isotonic SF, patients with open fracture were given tetanus prophylaxis and 3×1 antibiotic treatment with Cefazolin-Na 1 g for 3 days, and NSAID was started. Cefazolin-Na 1 g antibiotic prophylaxis was applied to the patients, 1 dose before surgery and 2 doses after surgery. The patients were operated under spinal anesthesia by applying a tourniquet in the supine position.

## 2.2.1. Single stage surgery

**Figure 1:**
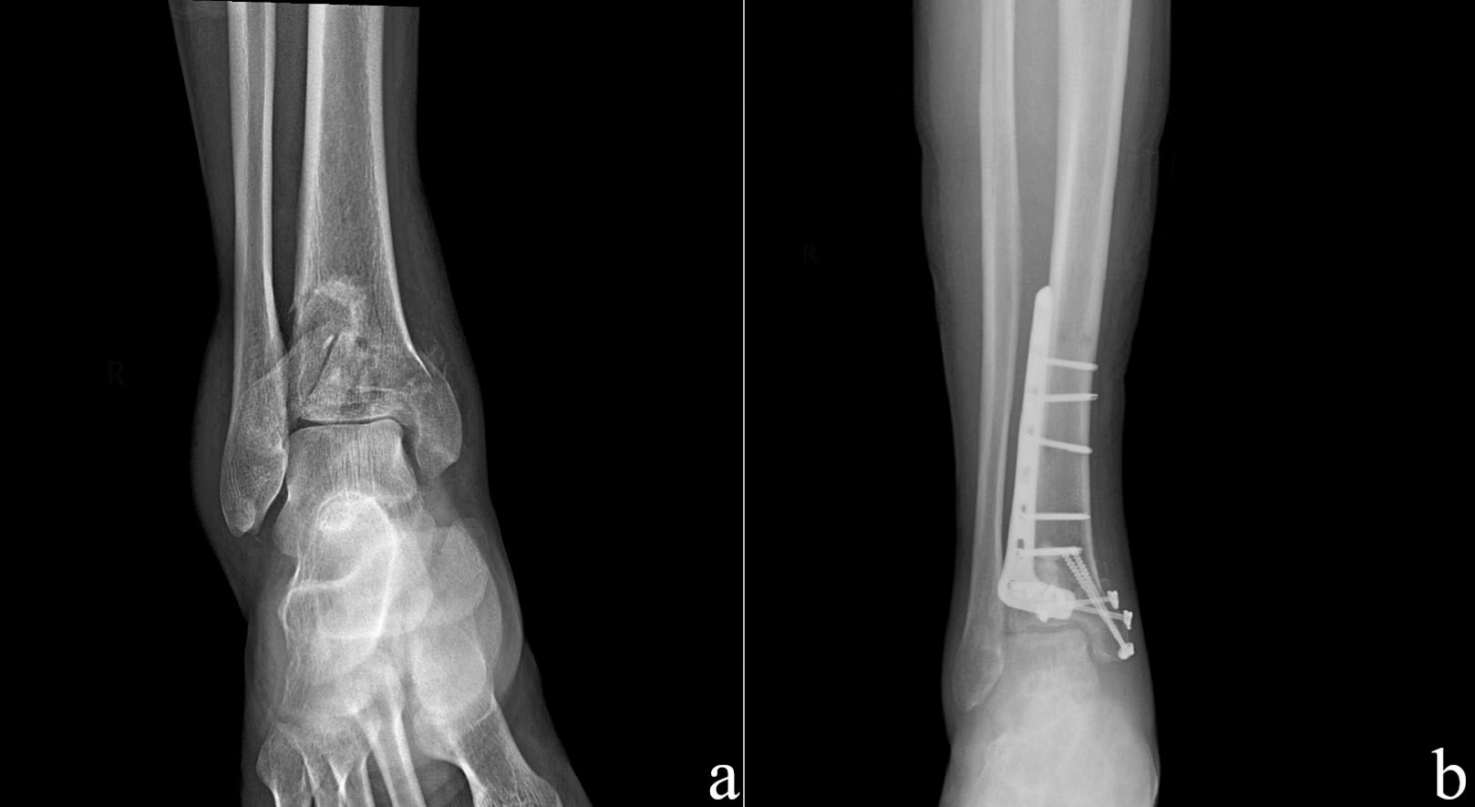
Preoperative (a) and postoperative (b) x-ray images of single step surgery.

Patients who were admitted to the emergency room early, had low soft tissue edema, had no preoperative disease, did not use anticoagulants were operated for the first 24 h. In patients without a complex fibula fracture (fragmented or segmental fracture), the fibula was fixed with a 1/3 tubular plate by entering through the ankle laterally through a longitidunal incision. Patients with complex fibula fractures were flicked after reduction of the tibia. The distal tibia was reached using an anteromedial incision. The tibialis anterior tendon was preserved. After reaching the fracture line and cleaning the fracture hematoma, the length of the extremity was achieved under manual traction. The fragments were then temporarily fixed with K wires after the best possible reduction of fragments. Metaphyseal regions with defects were filled with allografts. The fracture was fixed using either a 3.5 mm locked distal tibia anatomical plate or a 3.5 mm locked anteromedial pilon plate and, if necessary, canulated screws were used (Figures 1a and 1b). Joint face restoration, plaque orientation and fracture fixation were checked by fluoroscopy. At the end of each surgery, the anatomical sequence was checked AP-side plan. The operation area of the patients with no problems was washed with 5000 cc of saline and the wound site was sutured with 2-0 Ethicon (Ethicon Inc., Somerville, NJ, USA) and closed. No drains were used in patients.

## 2.2.2. Double stage surgery

**Figure 2:**
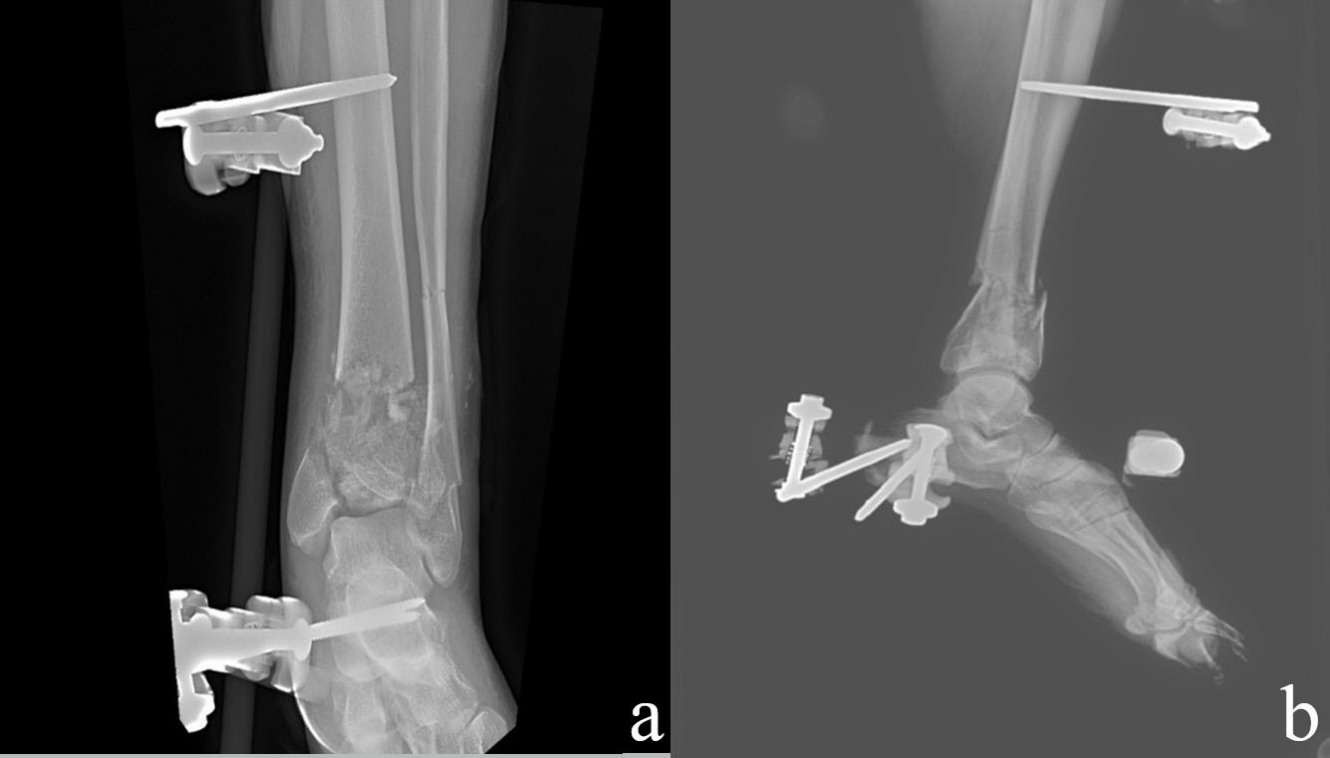
Anteroposterior (a) and lateral (b) x-ray images after external fixation (first step of two stage surgery).

**Figure 3:**
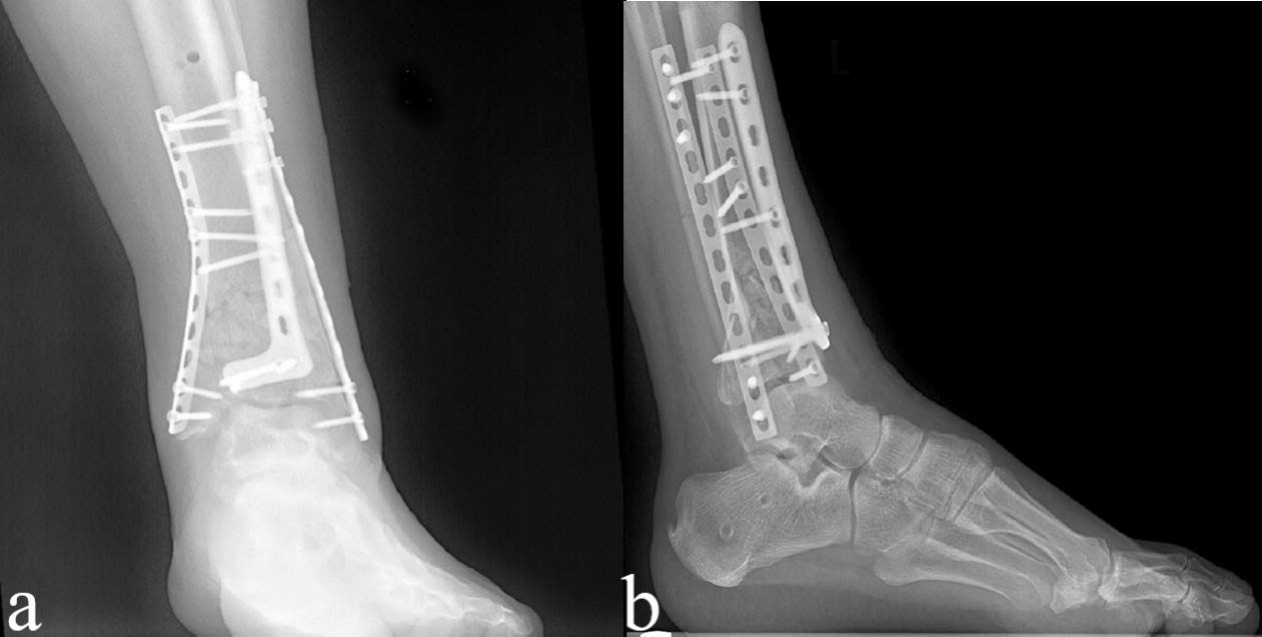
Anteroposterior (a) and lateral (b) x-ray images after ORIF (second step of two stage surgery).

In patients with severe edema in soft tissue and open fractures, two-stage surgical treatment was planned and tibio-calcaneal external fixator was applied within the first 24 h (Figures 2a and 2b). Under fluoroscopy, the fracture line was distracted and the best possible reduction was made and the length of the limb was achieved. External fixator was placed by inserting transmission screws to pass through 2 tibia and 1 or 2 calcaneus. The patients were left to the second operation without interfering with the fibula fractures. After monitoring the edema in the ankle and seeing that there was no circulatory problem, the patients were discharged with dvt prophylaxis and were taken to the weekly outpatient control. After the external fixators of the patients, whose edema in the ankle decreased, the prick test was positive and suitable for the operation, were removed under sterile conditions, ORIF was performed as in single-stage surgical treatment (Figures 3a and 3b).

## 2.2.3. Ilizarov

The patients were fixed firstby applying ORIF in the fibula. Then, 2 rings are combined with 3 rods parallel to each other on the broken line and the leg is centered, leaving a safe soft tissue distance of at least 2 cm between the ring and the skin. For distal tibial fixation, 1 full ring was adjusted to be just above the ankle joint level. The frame was completed by adding half a ring to the foot from the back. The first upper ring is at least 5 cm distal from the knee joint level; the middle ring is fixed to the intact tibia close to the broken line by 2 pieces of 2 mm wire or 2 screws. The third middle distal ring was placed on the ankle by compressing the fracture with oliveliated K wires. After adjusting the axial aligment, rotation, leg length, it was fixed using 3 rods with the other two rings above. The most distal half-ring was placed to support the forefoot to protect the reduction of the fracture line and to prevent equinovarus contractures. They were checked with fluoroscopy.

After the surgery, all patients were routinely treated with elevations, elastic bandages and cold treatments to the extremity. Postoperative first 24 h, cefazolin-sodium 3×1 gr parenteral (I.V.) antibiotic prophylaxis was applied and then the antibiotic therapy was stopped. None of the patients were pressed on their feet during the acute period. Patients without clinical and wound location problems were discharged with short leg splint support (except for patients treated with Ilizarov). Patients with no wound problems had their stitches removed for 15th days. Patients were given passive and active knee and ankle exercises from 30th day. Patients were called to a monthly checkup and evaluated clinically and radiologically. Patients were checked for compliance with the recommended rehabilitation program. During the clinical examination, ankle and knee movements were evaluated and radiologically two-way ankle and mortis were graphed. Collapse, loss of reduction, formation of callus tissue were evaluated. In the anteroposterior and lateral radiographs, the formation of callus was at a level that would allow the patient to give full load and the loss of the fracture line was considered as full recovery [12]. Partial load was started to be given to the patients after 45 days. Full load was started after 12–16 months of clinical and radiological improvement.

## 2.3. Statistical analysis

SPSS 18.0 version (SPSS, Inc., Chicago, IL, USA) program was used for statistical analysis of the study. Descriptive data were given as mean ± standard deviation, number or percentage. Categorical variables were compared using the chi-square test or Fischer’s exact test. Pairwise comparisons were made using the Mann–Whitney U test and evaluated using Bonferroni correction. The statistically significant level was set at P < 0.05.

## 3. Results

The demographic characteristics of the patients evaluated, the type of fracture according to the Rüedi/Allgöwer classification and information about the mechanism of trauma are given in Table 1. The mean age of the patients was 42.5 ± 14.4 (20–82) and the mean follow-up was 36.8 months (15–55). 44 of the patients had right (50.5%), 41 had left (47.1%), and 2 (2.2%) patients had bilateral pilon fractures. There were 7 patients with type 1 open fractures and all open fractures were treated with two step surgery.

**Table 1:** Demographic features of patients, Rüedi-Allgöwer classification, mechanism of the trauma and complications.

	One step surgery	Two step surgery	Ilizarov	Total
Number of patients	38 (43.6%)	26 (29.8%)	23 (26.4%)	87 (100%)
Sex				
Female	8(9.1%)	5 (5.7%)	4 (4.5%)	17 (19.5%)
Male	30 (34.4%)	21 (24.1%)	19 (21.8%)	70 (80.4%)
Rüedi-Allgöwer classification				
Type-1	7 (7.8%)	0 (0 %)	0 (0 %)	7 (7.8%)
Type-2	12 (13.4%)	8 (8.9%)	4 (4.4 %)	24 (26.9%)
Type-3	19 (21.3%)	20 (22.4%)	19 (21.3%)	58 (65.1%)
Trauma mechanism				
Fall	20 (22.9%)	16 (18.3%)	16 (18.3%)	54 (62%)
Traffic accident	14 (16%)	8 (9.1%)	6 (6.8%)	28 (32.1%)
Other reasons	3 (3.4%)	1 (1.1%)	1 (1.1 %)	5 (22.9%)
Smoking	16 (18.3%)	12 (13.7%)	11(12.6%)	49 (56.3%)
Diabetes mellitus	14 (16%)	10 (11.4%)	9 (10.3%)	33 (37.9%)
Skin complication	6 (6.8%)	0 (0%)	0 (0%)	6 (6.8%)
İnfection	2 (2.2%)	0 (0%)	0 (0%)	2 (2.2%)

The average AOFAS score of the patients included in the whole study was 77.6 ± 15.6 (mild). The AOFAS functional results according to the surgical tecnique are given in Table 2. When the AOFAS results of the groups were compared with each other, there was no statistically significant difference between the groups (P = 0.880). According to this result, there was no difference in terms of ankle functional scores after all 3 surgical treatments. 

While skin complication was not seen in our patients who underwent two-stage surgery and Ilizarov, it developed in 6 (15.7%) of our patients who underwent single-stage surgery. It was determined that 4 of these patients had diabetes mellitus (DM) and 3 of 6 patients smoked. The skin defects of 4 patients with skin complications were treated by grafting by plastic surgery. Superficial wound problems developed in our other 2 patients were treated with appropriate wound care. In one of our patients who developed skin defects, the plaque was removed and treated with plaster at the 3rd postoperative month because the infection could not be controlled. 

Functional results of the patients according to the type of fracture are given in Table 3. When AOFAS results were evaluated for the fracture type, a difference was observed between the groups (P = 0.004). While there was no difference between the results of type 1 and type 2 fractures, a statistically significant AOFAS score was found in type 3 fractures. It shows that lower functional results are obtained especially in type 3 fractures, and the functional results of more complicated fractures have also deteriorated.

**Table 2:** AOFAS results according to the surgical technic.

AOFAS results	One stage surgery	Two stage surgery	Ilizarov	P value
Excellent	1 (2.6%)	1 (3.5%)	0 (0%)	P = 0.880
Good	12 (31.5%)	7 (25%)	6 (26%)	
Mild	11 (28.9%)	5 (17.8%)	8 (34.7%)	
Poor	14 (36.8%)	15 (53.5%)	9 (39.1%)	
Total	38 (%100)	28 (100%)	23 (100%)	

**Table 3:** Functional results according to the type of fracture.

AOFAS results	Type-1 fracture	Type-2 fracture	Type-3 fracture	P value
Excellent	0	2 (2.2%)	0	P = 0.004*
Good	3 (3.4%)	11 (12.4%)	11 (12.4%)	
Mild	2 (2.2%)	8 (9%)	14 (15.7%)	
Poor	2 (2.2%)	3 (3.4%)	33 (37.1%)	
Total	7 (%100)	24 (100%)	58 (100%)	

* Results of type 3 fractures are significantly poor when compared types 1 and 2.

## 4. Discussion

The most striking result in the evaluations made at the end of our study is that, despite the different surgical approaches and implants, there is no difference between ankle functional results of the patients. However, when analyzed by type of fractures, there is no difference between type 1 and type 2 fractures, whereas type 3 fractures have statistically significantly lower results in terms of ankle functional scores. This result made us think that functional results in pilon fractures are mostly related to fracture morphology at the time of initial trauma. Our other important result is that although different surgical approaches do not affect functional results, it is determined that especially early complications develop more in single-stage surgery.

In surgical pilon fractures, full improvement in ankle functions is generally not achieved. In the studies examining the postoperative ankle functions in the literature, the rate of good and excellent results varies, but is low. In the 35 pilon fractures they followed in Jansen et al. study, the average AOFAS score was 65 (bad) [13], Ketz et al. found the AOFAS score as 85.2 on average in 9 patients applying posterior plaque to the distal tibia, and 76.4 (medium) in 10 patients who operated with a standard anterior approach [14]. Zhao et al. reported that they achieved 81% good and excellent results in their studies using absorbable implants [15]. In our study, we did not find a significant difference in AOFAS scores in our patients who underwent different surgery. We observed that the ankle functional results did not change in all three treatment methods. In addition, we think that most of the cases (78%) were caused by axial compression (falling from height) and that the type 3 fracture rate was high in the lack of perfect results.

One of the early problems in pilon fracture surgery is soft tissue complications. Wound complications reaching 37% to 40% have been reported in the literature [16–17]. To reduce these soft tissue complications; After waiting for sufficient time after external fixation, techniques such as plating (2-stage treatment), treatment with indirect reduction techniques in possible fractures, using smaller and lower profile implants, avoiding anteromedial incision were used. In two-stage surgery; Patterson et al. [18] in the study, 0%, Sirkin et al. [19] reported skin complications in only one (3.4%) of the 30 closed pilon fractures in his study. In addition, Sands et al., using a lower profile implant (using a 3.5 mm clover plate for tibial fixation, 1/3 tubular plate or both), reported destructive skin complications in open pilon fractures in 14% (2 of 14 patients), and in closed pilon fractures in 4% (2 of 50 patients) [20]. In our study, 6 (15,4%) of our patients who underwent single-stage surgery developed destructive skin problems, while we did not encounter any destructive skin problems in our patients who underwent two-stage surgery and Ilizarov. We found that Ilizarov and two-stage surgical treatment is a more reliable method in terms of skin complications, in which our results are compatible with the literature.

Lack of long term complications such as ankle arthrosis and tibiotalar joint deformities, gait disturbance are limitations of our study.

Despite the different techniques applied to pilon fractures, it still remains a problem for orthopedists and patients. It should be remembered that the amount of axial compression at the time of the first trauma, the type of fracture and damage to the ankle joint are proportional to the prognosis. Although there are no differences in functional results, we think that the two-stage surgery or Ilizarov method is more successful considering the complications and one step surgery may cause wound problems. We think that better and satisfactory results can be achieved with more experienced orthopedists in specialized centers and the development of new treatment methods that will enable the joint cartilage to regenerate.

**Conflict of interest**

The authors have no conflict of interest to declare.
